# The efficacy of pancreatic juice cytology with liquid-based cytology for evaluating malignancy in patients with intraductal papillary mucinous neoplasm

**DOI:** 10.1186/s12876-020-01465-y

**Published:** 2020-09-29

**Authors:** Kazuya Miyamoto, Kazuyuki Matsumoto, Hironari Kato, Ryuichi Yoshida, Yuzo Umeda, Hirohumi Inoue, Takehiro Tanaka, Akihiro Matsumi, Yosuke Saragai, Yuki Fujii, Tatsuhiro Yamazaki, Daisuke Uchida, Takeshi Tomoda, Shigeru Horiguchi, Takahito Yagi, Hiroyuki Okada

**Affiliations:** 1 Department of Gastroenterology and Hepatology, Okayama University Graduate School of Medicine, Dentistry, and Pharmaceutical Science, 2-5-1 Shikata-cho, Okayama, 700-8558 Japan; 2 Department of Gastroenterological Surgery, Okayama University Graduate School of Medicine, Dentistry, and Pharmaceutical Science, Okayama, Japan; 3 Department of Pathology, Okayama University Graduate School of Medicine, Dentistry, and Pharmaceutical Science, Okayama, Japan

**Keywords:** IPMN, PJC, LBC, BD SurePath

## Abstract

**Background:**

Pancreatic juice cytology (PJC) is a tool for diagnosing malignant intraductal papillary mucinous neoplasm (IPMN); however, the accuracy is insufficient using the conventional method. Liquid-based cytology (LBC) improves the cell recovery rate, and almost all cells can be evaluated. We evaluated the efficacy of PJC with LBC for malignant IPMN.

**Methods:**

We retrospectively analyzed 90 patients with suspected malignant IPMN who underwent PJC before pancreatectomy. PJC with smear and LBC methods was conducted in 52 patients (between June 2003 to December 2011) and 38 patients (between January 2012 to December 2018). Based on the imaging studies, all of the patients were classified according to the international consensus guidelines for IPMN revised in 2017.

**Results:**

Of the 90 patients, 43 (48%) had malignant IPMN (high-grade dysplasia or invasive carcinoma), and the remaining patients had non-malignant IPMN (intermediate- or low-grade dysplasia). LBC increased the accuracy of PJC for the diagnosis of malignant IPMN (smear method: 56% [29/52] vs. LBC method: 76% [29/38]; *P* = 0.044). In a multivariate analysis, LBC was a significant factor influencing the accurate diagnosis of PJC (odds ratio: 3.52; *P* = 0.021). Furthermore, LBC increased the accuracy of PJC for malignant IPMN in patients with worrisome features (smear method: 66% [19/29] vs. LBC method: 93% [14/15]; *P* = 0.043).

**Conclusions:**

LBC increases the accuracy of PJC for diagnosing malignant IPMN compared with the conventional smear method.

## Background

The increased detection of pancreatic cysts due to improvements in imaging studies has led to a surge in interest in intraductal papillary mucinous neoplasm (IPMN). Most IPMNs are benign, but some can progress to malignant IPMNs. However, it remains difficult to distinguish malignant IPMN from benign IPMN.

According to the international consensus guidelines for IPMN revised in 2017 [1], high-risk stigmata (HRS) were considered a recommended indicator for resection, and worrisome features (WF) were considered a recommended indicator for further examinations. However, the rate of malignant IPMN in patients with HRS and WF were reported to be 49–57% and 16–27%, respectively [2, 3]. Although the presence of mural nodules (MN) on endoscopic ultrasonography (EUS) has been shown to be a good predictor of malignant branch duct IPMN (BD-IPMN) [4–10], about 10% of malignant BD-IPMNs have been reported in patients without MN [8, 11, 12]. As mentioned above, the diagnostic ability of the imaging studies for malignant IPMN is limited.

Pancreatic juice cytology (PJC) under endoscopic retrograde cholangiopancreatography (ERCP) is an examination for IPMNs, and suspicion of malignancy or positive malignancy by PJC is an absolute indication for resection. However, the sensitivity of PJC for malignant IPMN was found to be only 35% in a meta-analysis [13], and the accuracy was also unsatisfactory (33–64%) [2, 14, 15]. Furthermore, the incidence of post-ERCP pancreatitis (PEP) was also reported to be about 8–25% [2, 14–16]. These are challenges that must be considered when performing PJC for the diagnosis of malignant IPMN.

Liquid-based cytology (LBC), developed in 1991 [17], was originally used in cervical cytology and has since been widely applied to various organs. LBC has the following benefits: (1) it allows for the efficient transfer of cells from the collecting device, and almost all cells can be analyzed; (2) by using separation reagents, it is possible to selectively reduce red blood cells, inflammatory cells and mucus—in this way, the cells collected are mainly those required for the diagnosis, and unsatisfactory smears can be reduced; (3) the cellular materials are distributed evenly in one spot, leading to a reduction in screening time; and (4) using residual cellular materials, immunostaining or genetic tests can be conducted [18]. The effectiveness of LBC has been reported for some diseases [19–21]; however, no studies have described the application of LBC in IPMN patients.

Considering the benefits of LBC, it may be useful for the diagnosis of malignant IPMN with PJC. In the present study, we retrospectively investigated the efficacy of PJC using LBC for a malignant IPMN diagnosis.

## Methods

### Patients

A total of 104 patients with IPMNs who underwent PJC before pancreatectomy between June 2003 and December 2018 at Okayama University Hospital were retrospectively analyzed. PJC was basically performed for the patients with suspected malignant IPMN due to HRS or WF. PJC with the smear method was conducted between June 2003 and December 2011, while PJC with LBC was conducted between January 2012 and December 2018.

**Fig. 1:**
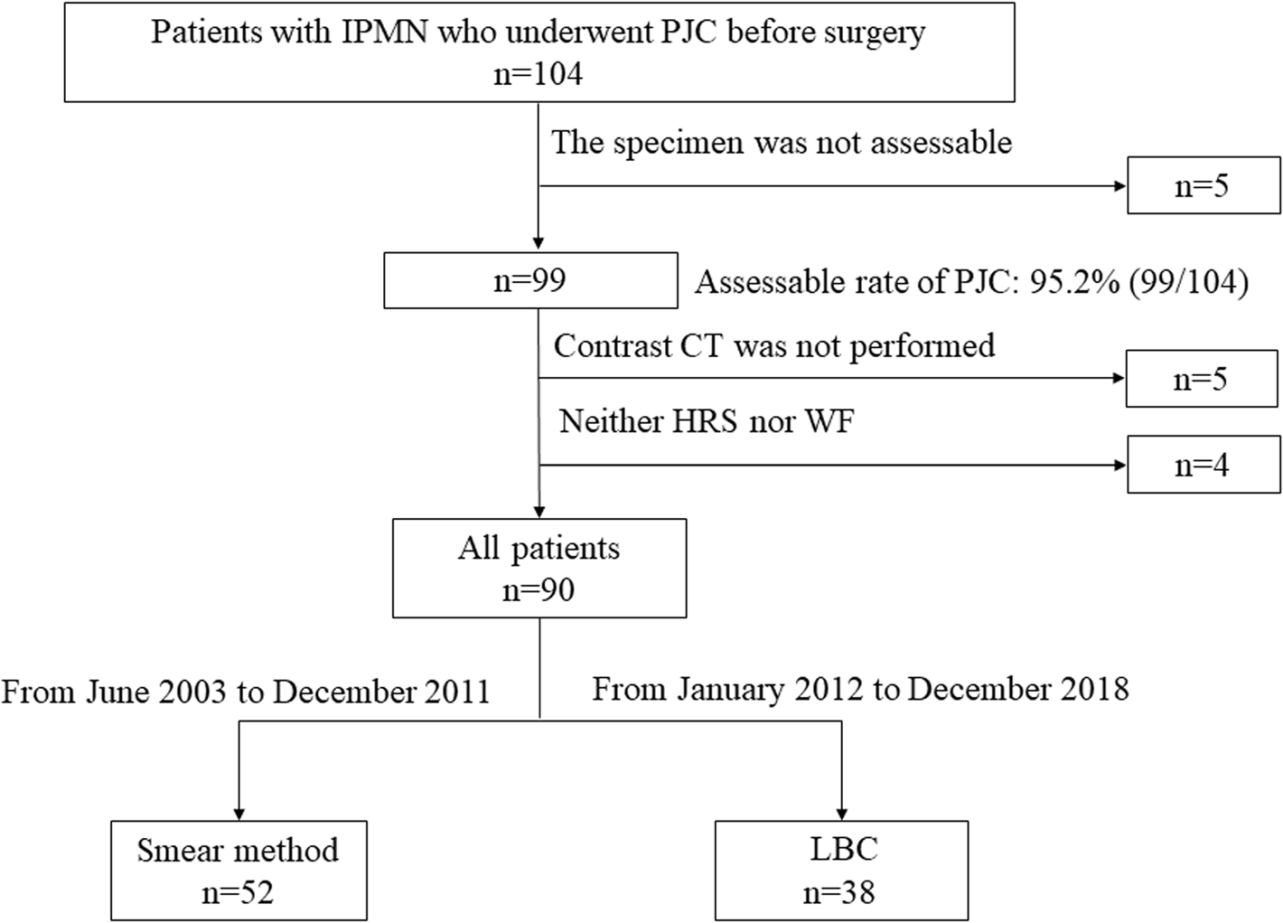
A flowchart for all patients of this study. *IPMN*, intraductal papillary mucinous neoplasm; *PJC*, pancreatic juice cytology; *CT*, computed tomography; *HRS*, high-risk stigmata; *WF*, worrisome features; *LBC*, liquid-based cytology

Figure 1 shows the study flow chart. Of the 104 patients with PJC, 5 lacked assessable specimens (smear method: 4, LBC method: 1). Another five patients who could not undergo contrast computed tomography (CT) because of a contrast agent allergy and four who did not satisfy the HRS or WF criteria were excluded from this study. Ultimately, 90 patients (52 patients with the smear method, 38 patients with LBC) were analyzed. Before endoscopic retrograde cholangiopancreatography (ERCP), informed consent was obtained from all patients.

The study was approved by the Okayama University School of Medicine Clinical Ethics Committee on Human Experiments in accordance with the Declaration of Helsinki (Approved number:1904–028).

## Methods

### The imaging diagnosis

For the imaging studies, all patients underwent EUS and either or both magnetic resonance cholangiopancreatography (MRCP) or enhanced CT. The cystic size and diameter of the main pancreatic duct were evaluated by MRCP or enhanced CT, while the presence and height of mural nodules were evaluated by EUS or enhanced CT. The height of mural nodules was defined as the vertical distance from the septum to the top of the mural nodule [22]. Based on the imaging studies, all of the patients were classified as HRS or WF according to the international consensus guidelines for IPMN revised in 2017 [1].

### ERCP procedures and collecting pancreatic juice

ERCP was performed for the patients in a prone or semi-prone position under conscious sedation using intravenous diazepam and pethidine hydrochloride with CO_2_ insufflation. Pancreatography was carried out using a duodenoscope (JF260v; Olympus, Tokyo, Japan), and pancreatic duct cannulation was performed using a long, tapered catheter (PR-220Q; Olympus) with a 0.025-in. guidewire (VisiGlide or VisiGlide2; Olympus) or a 0.035-in. guidwire (RevoWave; Piolax, Kanagawa, Japan). After cannulation, the cannula was changed to a sampling catheter with a side hole (PR-130Q; Olympus) or a 6-Fr uneven double-lumen catheter (UDC; Piolax) with saline washing. Sampling of the pancreatic juice was carried out with negative pressure using a 10 mL syringe for about 5 min. A serial pancreatic-juice aspiration cytologic examination (SPACE) was performed using a 5-French endoscopic naso-pancreatic drainage (ENPD) tube (Nasal. Pancreatic Drainage Set; Cook Medical, Japan). In patients with ENPD placement, PJC via an ENPD tube was able to be repeatedly performed, and PJC was usually performed 4–5 times over 3–4 days. The diagnosis of PEP and the severity of PEP were judged according to the Cotton criteria [23]. PEP was defined as “clinical pancreatitis with an elevated serum amylase level >3 times the upper limit of normal after more than 24 h.” The severity of PEP was as follows: mild, requiring admission or prolongation of planned admission to 2–3 days; moderate, requiring hospitalization of 4–10 days; and severe, requiring hospitalization for more than 10 days or hemorrhagic pancreatitis, phlegmon, pseudocyst, or intervention (percutaneous drainage or surgery).

### Preparation of the LBC sample

**Fig. 2:**
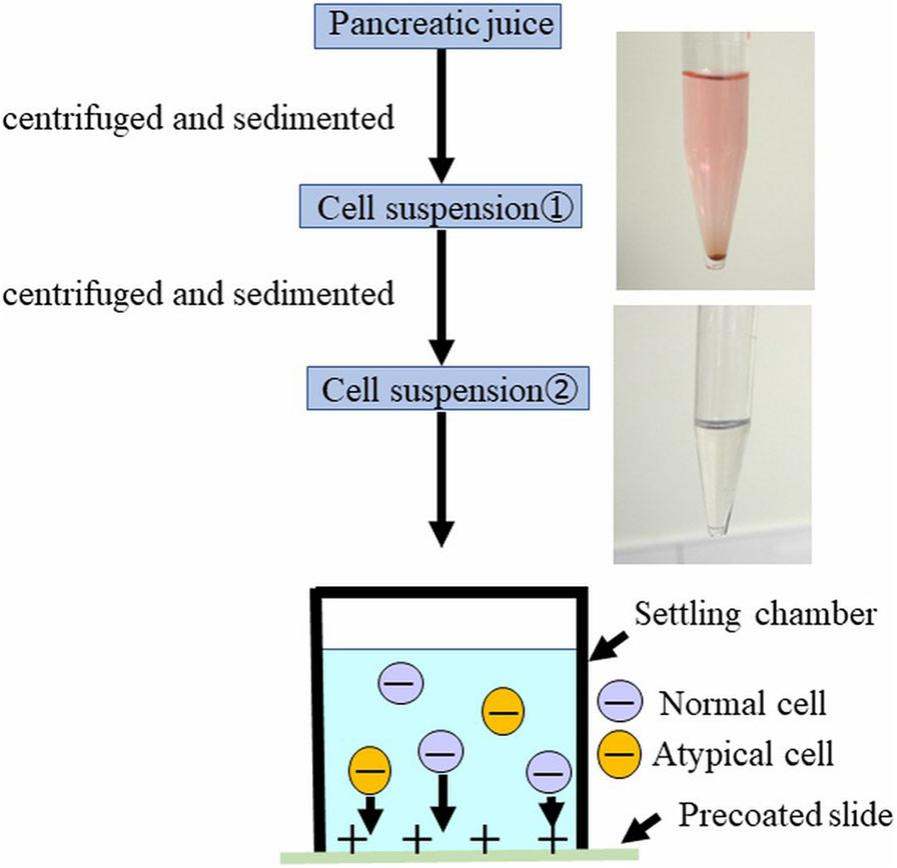
Preparation of the LBC sample with a schematic illustration. First, pancreatic juice was centrifuged, and the supernatant was discarded. The material was then suspended in fixative (CytoRich™ RED). Next, the suspension was centrifuged, and the supernatant was discarded. The material was suspended in distilled water. Third, the cell suspension was poured onto a precoated slide equipped with a settling chamber. The cells became attached to the slide glass by the charge and gravity. *LBC*, liquid-based cytology

**Fig. 3:**
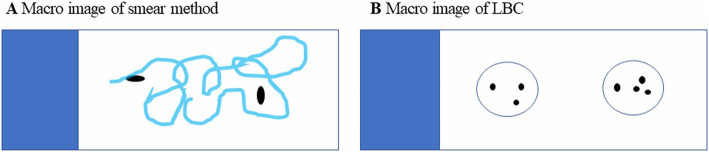
Macro images of the slide glasses with a schematic illustration: (**a**) Macro image of smear method. (**b**) Macro image of LBC. With LBC, the cellular materials were distributed in two spots. *LBC*, liquid-based cytology

**Fig. 4:**
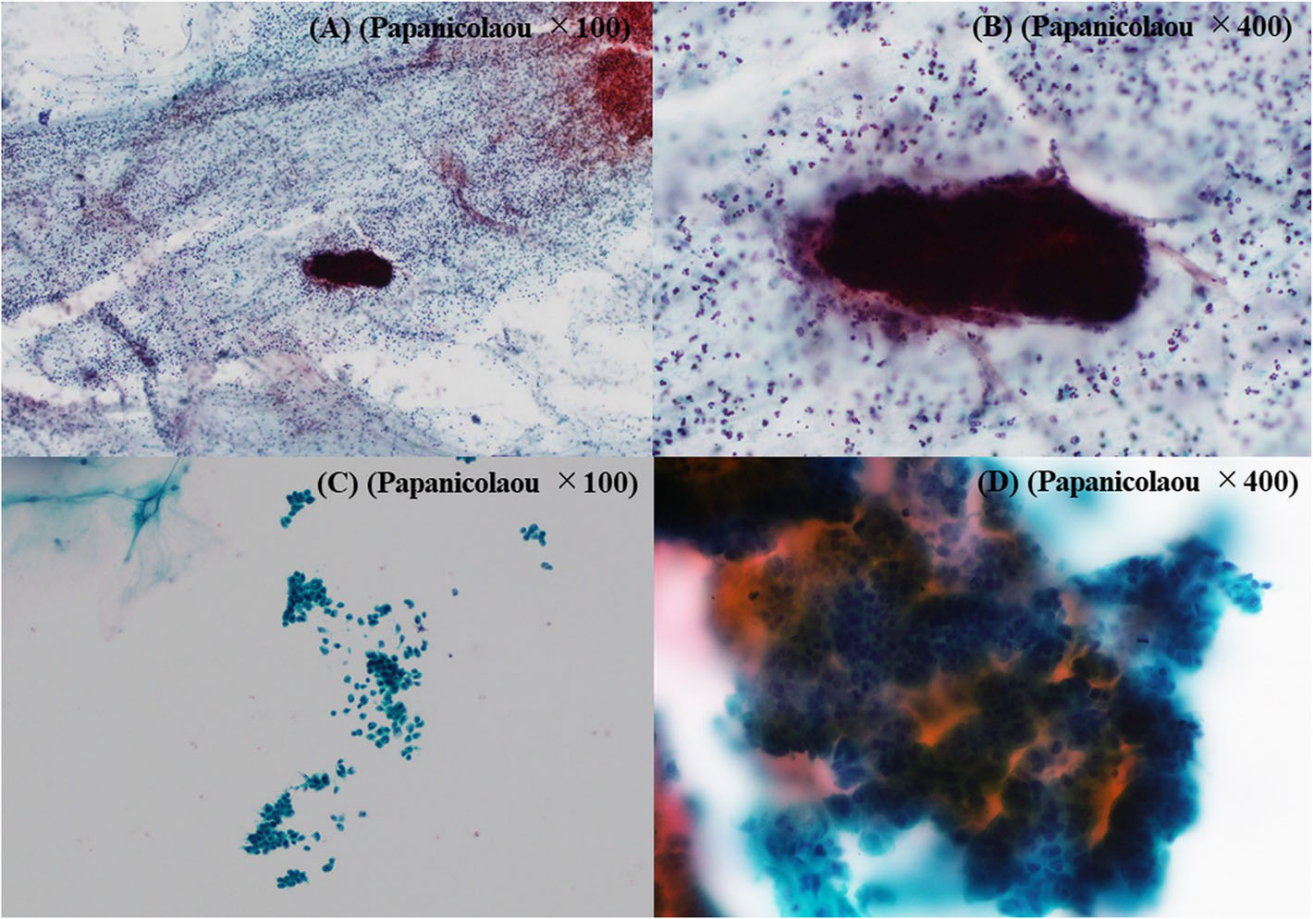
PJC findings of malignant IPMN: **a** A low-power-field image of the smear method. Many inflammatory cells and a large amount of mucus were found in the background, and the tumor cell cluster was heavily stained. **b** A high-power-field image of the smear method. It was difficult to evaluate the cells at the margin of the tumor cell cluster because of overlapping. **c** A low-power-field image of LBC. There were few overlapping cells. The background of inflammatory cells and mucus was removed, so the tumor cells scattered solitarily could be evaluated. **d** A high-power-field image of LBC. The cells at the margin of the tumor cell cluster could be evaluated. *PJC*, pancreatic juice cytology; *IPMN*, intraductal papillary mucinous neoplasm; *LBC*, liquid-based cytology

Figure 2 shows the preparation of the LBC sample with a schematic illustration. To process LBC samples, a BD SurePath (Nippon Becton Dickinson Company, Tokyo, Japan) was used. Pancreatic juice was centrifuged, and the supernatant fluid was discarded. The material was suspended in fixative (CytoRich RED; Nippon Becton Dickinson Company, Tokyo, Japan), and a cell suspension was prepared. The suspension was centrifuged, and the supernatant was discarded. The material was then resuspended in distilled water, and the cell suspension was poured into a precoat slide equipped with a settling chamber. The cell surface was negatively charged while the precoated slide was positively charged, and the cells became attached to the slide glass using the charge and gravity. Figure 3 shows macro images of the slide glasses with a schematic illustration. Figure 4 shows the typical findings of the smear and LBC methods.

### Pathological diagnosis

All PJC specimens and the resected specimens were evaluated by two pathologists. The definition of malignancy with PJC was class IV-V according to the Papanicolaou classification system [24]. For the resected specimens, low- to intermediate-grade dysplasia (L-IGD) was determined to be benign, and high-grade dysplasia (HGD) and invasive carcinoma (IC) were determined to be malignant according to the 2010 WHO classification [25].

### Evaluating the endpoints

The primary endpoints were the comparison of the diagnostic ability of PJC for malignant IPMNs using LBC and conventional smear methods. The secondary endpoints were the evaluation of the factors contributing to the accuracy of PJC for the IPMN diagnosis and post-ERCP adverse events.

### Statistical analyses

All statistical analyses were evaluated using the JMP Pro software program, version 14 (SAS Institute, Cary, NC, USA). Pearson’s χ ^2^ test was used for the categorical variables, whereas the Mann-Whitney *U* test was used for continuous data. A multivariate analysis was performed using logistic regression in order to extract significant factors contributing to the accuracy of a malignant IPMN diagnosis. Significant variables in the univariate analysis (*P* < 0.2) were selected for inclusion in the multivariate model. The significance level was set at *P* < 0.05.

## Results

### Patient characteristics

The median age was 70 years old (interquartile range [IQR]: 65–74 years old). The types of IPMN were as follows: BD-IPMN, 33 (37%) patients; mixed IPMN, 43 (48%) patients; and main duct (MD)-IPMN, 14 (16%) patients. Twelve (13%) patients underwent a SPACE. A SPACE was performed more frequently in the LBC group than in the smear method group (Table 1) (smear method: 1/52, 2% vs. LBC: 11/38, 29%; *P* < 0.001).

**Table 1:** Clinicopathologic features of 90 patients with resected IPMNs

	All patients (*n* = 90)	Smear method (*n* = 52)	LBC (*n* = 38)	*P* value
Age (IQR), years	70 (65–74)	69 (64–73)	72 (67–76)	0.084
Sex (male/female)	59/31	37/15	22/16	0.191
Location of main lesion (Ph/Pbt)	50/40	30/22	20/18	0.633
Type of IPMN, n (%)				0.075
BD	33 (37)	21 (40)	12 (32)	
Mixed	43 (48)	20 (38)	23 (61)	
MD	14 (16)	11 (21)	3 (8)	
Pathological diagnosis, n (%)				0.626
L-IGD	47 (52)	24 (46)	23 (61)	
HGD	15 (17)	10 (19)	5 (13)	
IC	28 (31)	18 (35)	10 (26)	
Diameter of dilated branch duct (IQR), mm	27.0 (17.0–40.3)	30.0 (11.0–42.5)	26.0 (19.8–36.8)	0.990
≥ 30 mm, n (%)	41 (46)	27 (52)	14 (37)	0.156
Diameter of MPD (IQR), mm	6.0 (4.0–10.0)	6.0 (4.1–10.0)	6.4 (4.0–10.0)	0.928
5–9 mm, n (%)	35 (39)	20 (38)	15 (39)	0.923
≥ 10 mm, n (%)	23 (26)	14 (27)	9 (24)	0.728
Patients with MN, n (%)	67 (74)	39 (75)	28 (74)	0.888
Height of MN (IQR), mm	7.0 (4.0–15.0)	7.0 (4.0–13.0)	9.5 (4.0–17.5)	0.366
Enhancing MN ≥ 5 mm, n (%)	31 (46)	15 (38)	16 (57)	0.130
SPACE, n (%)	12 (13)	1 (2)	11 (29)	< .001

*IPMN*, intraductal papillary mucinous neoplasm; *LBC*, liquid-based cytology; *IQR*, interquartile range; *Ph*, pancreatic head; *Pbt*, pancreatic body and tail; *BD,* branch duct; *MD*, main duct; *L-IGD*, low-to intermediate-grade dysplasia; *HGD*, high-grade dysplasia; *IC*, invasive cancer; *MPD*, main pancreatic duct; *MN*, mural nodule; *SPACE*, serial pancreatic juice aspiration cytological examination

### Diagnostic ability of PJC for malignant IPMNs

Table 2 shows the diagnostic ability of PJC for malignant IPMNs in 90 patients. For all patients, the sensitivity, specificity and accuracy were 28, 98 and 64%, respectively. Compared to the smear method, the LBC showed an increased sensitivity and accuracy (sensitivity: 21 to 40%, accuracy: 56 to 76%). Furthermore, the accuracy with LBC was significantly greater than with the smear method (*P* = 0.044). The sensitivity and accuracy of PJC were increased by a SPACE (sensitivity: 26–50%, accuracy: 62–83%), although there were no significant differences (*P* = 0.301, *P* = 0.142).

**Table 2:** Diagnostic ability of PJC for malignant IPMNs

		Sensitivity, (%)	Specificity, (%)	PPV, (%)	NPV, (%)	Accuracy, (%)
Smear method	(n = 52)	21 (6/28)	96 (23/24)	86 (6/7)	51 (23/45)	56 (29/52)
LBC	(n = 38)	40 (6/15)	100 (23/23)	100 (6/6)	72 (23/32)	76 (29/38)
All patients	(n = 90)	28 (12/43)	98 (46/47)	92 (12/13)	60 (46/77)	64 (58/90)

*PJC* pancreatic juice cytology, *IPMN* intraductal papillary mucinous neoplasm, *PPV* positive predictive value, *NPV* negative predictive value; LBC, liquid-based cytology

### Evaluating the factors contributing to the accuracy of PJC for the IPMN diagnosis

Table 3 summarizes the results of the univariate and multivariate analyses for factors contributing to the accuracy of PJC for the IPMN diagnosis. In the univariate analysis, LBC (*P* = 0.044), SPACE (*P* = 0.142), enhancing MN > 5 mm (*P* = 0.168), diameter of dilated branch duct ≥ 3 cm (*P* = 0.043), lymphadenopathy (*P* = 0.092) and an increased serum level of CA19–9 (*P* = 0.002) were significant factors (*P* < 0.2). In the multivariate analysis, LBC (odds ratio [OR]: 4.25; 95% CI, 1.24–14.54; *P* = 0.021), enhancing MN > 5 mm (OR: 0.23; 95% CI, 0.07–0.73; *P* = 0.013), diameter of dilated branch duct ≥ 3 cm (OR: 4.41; 95% CI, 1.45–13.35; *P* = 0.009) and increasing serum level of CA19–9 (OR: 0.24; 95% CI, 0.07–0.79; *P* = 0.019) were concluded to be significant factors contributing to the accuracy of PJC for the IPMN diagnosis.

**Table 3:** Results of univariate and multivariate analyses for factors contributing to the accuracy of PJC for the IPMN diagnosis (n = 90)

			Univariate	Multivariate		
Factors	n	Ratio of malignancy, n (%)	*P* value	Odds ratio	95% CI	*P* value
Age, ≥ 70 years old	48	26 (54)	0.977			
Sex, male	59	27 (46)	0.992			
Location of main lesion, Ph	50	23 (46)	0.431			
MD or mixed-type, yes	57	31 (54)	0.212			
LBC, yes	38	15 (39)	0.044	4.25	1.24–14.54	0.021
SPACE, yes	12	4 (33)	0.142	2.04	0.31–13.28	0.455
Obstructive jaundice, yes	6	6 (100)	0.444			
Enhancing MN ≥ 5 mm, yes	31	19 (61)	0.168	0.23	0.07–0.73	0.013
Diameter of MPD ≥ 10 mm, yes	23	14 (61)	0.358			
Past history of pancreatitis, yes	10	4 (40)	0.697			
Diameter of dilated branch duct ≥3 cm, yes	41	15 (37)	0.043	4.41	1.45–13.35	0.009
Thickness and/or enhancing cyst walls, yes	26	10 (38)	0.276			
Abrupt change in caliber of pancreatic duct with distal pancreatic atrophy, yes	22	16 (73)	0.927			
Lymphoadenopathy, yes	4	4 (100)	0.092	0.15	0.01–2.12	0.159
Increasing serum level of CA19–9, yes	22	18 (82)	0.002	0.24	0.07–0.79	0.019
Increasing size of cyst over 5 mm/2 years, yes	15	8 (53)	0.617			

*IPMN*, intraductal papillary mucinous neoplasm; *CI*, confidence interval; *Ph*, pancreatic head; *MD*, main duct; *LBC*, liquid-based cytology; *SPACE*, serial pancreatic juice aspiration cytological examination; *MN*, mural nodule; *MPD*, main pancreatic duct; *CA19–9*, carbohydrate antigen 19–9

### The evaluation for HRS and WF patients

**Fig. 5:**
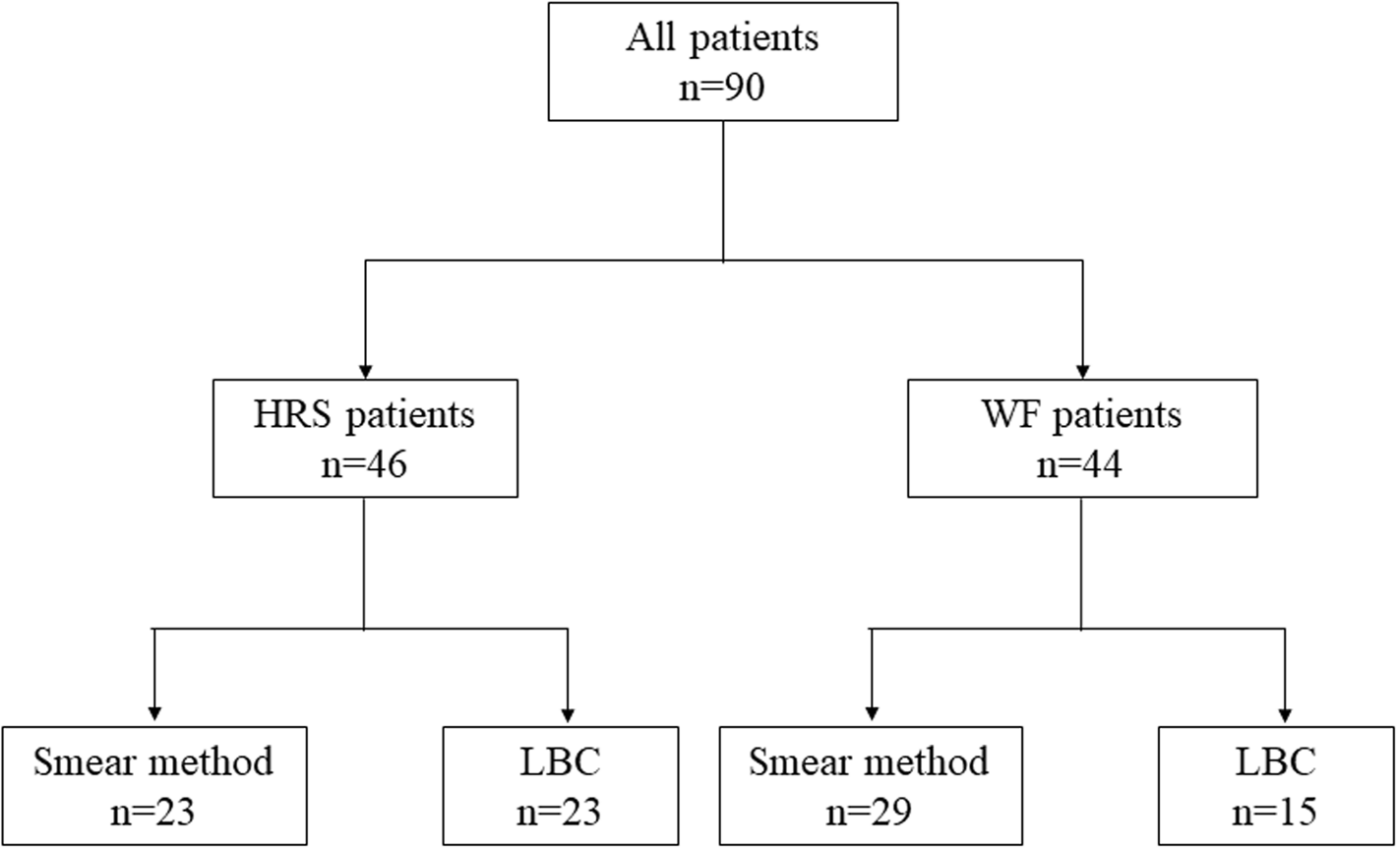
A flowchart for HRS and WF patients in this study. *HRS*, high-risk stigmata; *WF*, worrisome features; *LBC*, liquid-based cytology

Figure 5 shows the flowchart of HRS and WF patients. Forty-six patients with HRS were evaluated separately before and after LBC introduction (smear method: 23, LBC: 23). Forty-four patients with WF were also evaluated separately (smear method: 29, LBC: 15).

Tables 4 and 5 summarize the clinicopathologic features of the 46 patients with HRS and the 44 patients with WF, respectively. Among the HRS patients, the median age was 71 years old (IQR: 66–75 years old). Six (13%) patients had obstructive jaundice. An MPD diameter ≥  10 mm was found in 23 (50%) patients. Enhancing MN > 5 mm was found in 31 (72%) patients. Six (13%) patients underwent SPACE. There were no patients who underwent a SPACE in the smear method group, and a SPACE was performed significantly more frequently in the LBC group than in the smear method group.

**Table 4:** Clinicopathologic features of 46 patients with resected IPMNs (HRS patients)

	All patients (*n* = 46)	Smear method (*n* = 23)	LBC (n = 23)	*P* value
Age (IQR), years	71 (66–75)	70 (66–73)	71 (66–77)	0.733
Sex (male/female)	27/19	15/8	12/11	0.369
Location of main lesion (Ph/Pbt)	21/25	11/12	10/13	0.767
Type of IPMN, n (%)				0.128
BD	9 (20)	4 (17)	5 (22)	
Mixed	25 (54)	10 (43)	15 (65)	
MD	12 (26)	9 (39)	3 (13)	
Pathological diagnosis, n (%)				0.407
L-IGD	16 (35)	6 (26)	10 (43)	
HGD	10 (22)	5 (22)	5 (22)	
IC	20 (43)	12 (52)	8 (35)	
Obstructive jaundice, n (%)	6 (13)	3 (13)	3 (13)	1.000
Diameter of dilated branch duct (IQR), mm	25.5 (6.0–42.3)	17.0 (0–46.0)	28.0(20.0–42.0)	0.370
≥ 30 mm, n (%)	21 (46)	10 (44)	11 (48)	0.767
Diameter of MPD (IQR), mm	10.0 (5.0–12.0)	10.0 (6.0–13.0)	8.0 (5.0–12.0)	0.205
≥ 10 mm, n (%)	23 (50)	14 (61)	9 (39)	0.140
Patients with MN, n (%)	43 (93)	22 (96)	21 (91)	0.550
Height of MN (IQR), mm	12.0 (7.0–18.0)	11.0 (7.0–17.3)	13.0 (7.5–19.5)	0.626
Enhancing MN ≥ 5 mm, n (%)	31 (72)	15 (68)	16 (76)	0.558
SPACE, n (%)	6 (13)	0 (0)	6 (26)	0.009

*IPMN*, intraductal papillary mucinous neoplasm; *HRS*, high-risk stigmata; *LBC*, liquid-based cytology; *IQR*, interquartile range; *Ph*, pancreatic head; *Pbt*, pancreatic body and tail; *BD,* branch duct; *MD*, main duct; *L-IGD*, low-to intermediate-grade dysplasia; *HGD*, high-grade dysplasia; *IC*, invasive cancer; *MPD*, main pancreatic duct; *MN*, mural nodule; *SPACE*, serial pancreatic juice aspiration cytological examination

**Table 5:** Clinicopathologic features of 44 patients with resected IPMNs (WF patients)

	All patients (*n* = 44)	Smear method (*n* = 29)	LBC (*n* = 15)	*P* value
Age (IQR), years	69 (63–74)	68 (61–72)	74 (68–76)	0.135
Sex (male/female)	32/12	22/7	10/5	0.516
Location of main lesion (Ph/Pbt)	29/15	19/10	10/5	0.939
Type of IPMN, n (%)				0.341
BD	24 (55)	17 (59)	7 (47)	
Mixed	18 (41)	10 (34)	8 (53)	
MD	2 (5)	2 (7)	0 (0)	
Pathological diagnosis, n (%)				0.105
L-IGD	31 (70)	18 (62)	13 (87)	
HGD	5 (11)	5 (17)	0 (0)	
IC	8 (18)	6 (21)	2 (13)	
Diameter of dilated branch duct (IQR), mm	28.0 (19.3–35.0)	30.0 (19.5–42.0)	23.0 (19.0–29.0)	0.725
≥ 30 mm, n (%)	20 (45)	17 (59)	3 (20)	0.015
Diameter of MPD (IQR), mm	5.0 (4.0–7.0)	5.0 (4.0–7.0)	5.0 (4.0–8.0)	0.542
5–9 mm, n (%)	22 (50)	14 (48)	8 (53)	0.751
Patients with MN, n (%)	24 (55)	17 (59)	7 (47)	0.450
Height of MN (IQR), mm	3.9 (3.0–4.8)	4.0 (3.0–6.0)	3.0 (1.0–4.0)	0.044
SPACE, n (%)	6 (14)	1 (3)	5 (33)	0.006

*IPMN*, intraductal papillary mucinous neoplasm; *WF*, worrisome features; *LBC*, liquid-based cytology; *IQR*, interquartile range; *Ph*, pancreatic head; *Pbt*, pancreatic body and tail; *BD*, branch duct; *MD*, main duct; *L-IGD*, low-to intermediate-grade dysplasia; *HGD*, high-grade dysplasia; *IC*, invasive cancer; *MPD*, main pancreatic duct; *MN*, mural nodule; *SPACE*, serial pancreatic juice. Aspiration cytological examination

Among the WF patients, the median age was 69 years old (IQR: 63–74 years old). A dilated branch duct diameter ≥ 3 cm was found in 20 (45%) patients. Twenty-four (55%) patients had detectable MN, and the median height of MN was 3.9 mm (IQR: 3.0–4.8 mm). Six (14%) patients underwent a SPACE. There were significant differences between the two groups in terms of the proportions of patients with a dilated branch duct diameter ≥ 3 cm, the median height of MN, and the proportion undergoing a SPACE.

### Diagnostic ability of PJC for malignant IPMNs in HRS and WF patients

Table 6 shows the diagnostic ability of PJC. For all patients, the sensitivity, specificity and accuracy were 33, 94 and 54% in patients with HRS and 15, 100 and 75% in patients with WF, respectively. In HRS patients, LBC showed a tendency toward an improvement in accuracy compared with smear method (smear method: 43% vs. LBC: 65%; *P* = 0.139). Furthermore, LBC significantly improved the accuracy compared with smear method in WF patients (smear method: 66% vs. LBC: 93%; *P* = 0.043).

**Table 6:** Diagnostic ability of PJC for malignant IPMNs (HRS and WF patients)

		Sensitivity, (%)	Specificity, (%)	PPV, (%)	NPV, (%)	Accuracy, (%)
HRS						
Smear method	(n = 23)	29 (5/17)	83 (5/6)	83 (5/6)	29 (5/17)	43 (10/23)
LBC	(n = 23)	38 (5/13)	100 (10/10)	100 (5/5)	56 (10/18)	65 (15/23)
All patients	(n = 46)	33 (10/30)	94 (15/16)	91 (10/11)	43 (15/35)	54 (25/46)
WF						
Smear method	(n = 29)	9 (1/11)	100 (18/18)	100 (1/1)	64 (18/28)	66 (19/29)
LBC	(n = 15)	50 (1/2)	100 (13/13)	100 (1/1)	93 (13/14)	93 (14/15)
All patients	(n = 44)	15 (2/13)	100 (31/31)	100 (2/2)	74 (31/42)	75 (33/44)

*PJC*, pancreatic juice cytology; *IPMN*, intraductal papillary mucinous neoplasm; *HRS*, high-risk stigmata; *WF*, worrisome features; *PPV*, positive predictive value; *NPV*, negative predictive value. *LBC*, liquid-based cytology

### Post-ERCP adverse events

Among the 104 patients who underwent ERCP for PJC, PEP developed in 13 (13%), and pneumonia developed in 1 (1.0%). The degree of severity of PEP was mild in 7 (6.7%) and moderate in 6 (5.8%); no severe cases of PEP occurred. All of the patients were treated with conservational therapy and improved. There were no patients who needed to cancel or delay their surgery due to the occurrence of PEP. Among the 104 patients, SPACE was performed in 14 (13%). Three of the 14 patients developed PEP (21%). There was no significant difference between the patients with and without a SPACE in terms of the frequency of PEP (SPACE: 21% vs. without SPACE: 11%; *P* = 0.278).

## Discussion

This study is the first to evaluate the efficacy of PJC using LBC with a large number of surgically resected IPMN cases. In our study, LBC increased the accuracy of PJC for a malignant IPMN diagnosis (smear method: 56% vs. LBC: 76%; *P* = 0.044) and proved to be a significant factor influencing an accurate diagnosis of PJC in the multivariate analysis (odds ratio [OR]: 3.52; *P* = 0.021). Second, LBC increased the accuracy of PJC particularly for diagnosing malignant IPMN in WF patients (smear method: 66% vs. LBC: 93%; *P* = 0.043).

The smear method has been used generally due to its convenience and low cost. However, issues associated with this method include the fact that the amount of cells placed on the slide glass varies depending on the skill of the operator, and dry denaturation causes poor cell preservation. Previous studies have reported that these issues accounted for two-thirds of cytological false negatives [26–28].

With LBC, all collected cells are placed in the fixative, and the cells required to make a diagnosis are mainly collected using separation reagents. The cell suspension is smeared uniformly onto two spots of a glass slide, with few overlapping cells, so the cell findings at the margin of the cluster can be analyzed. In addition, the background of inflammatory cells and mucus is removed, so the tumor cells scattered solitarily can be evaluated. Furthermore, because the scope of the speculum is narrowed, the speculum time is reduced. For these reasons, almost all cells can be efficiently analyzed. The LBC method is thus superior to the smear method with regard to assessing both the cellularity and cytomorphology. For the diagnosis of malignant IPMN, PJC has low sensitivity due to inadequate cellularity in most cases [29]. Using LBC, an increased sensitivity (21 to 40%) and negative predictive value (NPV) (51 to 72%) contributed to the increased accuracy of PJC.

Both the BD SurePath (Nippon Becton Dickinson Company, Tokyo, Japan) and ThinPrep (Hologic Japan, Tokyo, Japan) are commonly used LBC technologies in cervical cytology. These technologies differ by their methods of producing thin-layer slides. The BD SurePath uses no filters and employs a proprietary cell enrichment process that separates and reduces mucus, blood and inflammatory cells. The unnecessary debris is then trapped in a gradient density material that is removed. In contrast, the ThinPrep uses a membrane that controls the collection and transfer of diagnostic cells. Kenyon et al. reported that the addition of mucus did not reduce the cellularity with the BD SurePath; however, the cellularity was significantly reduced with ThinPrep [30]. They showed that direct obstruction of the filtration membrane of the ThinPrep due to excess mucus caused a reduction in the number of diagnostic cells. We therefore considered that the BD SurePath might be suitable for the diagnosis of malignant IPMN with rich mucus.

In patients with HRS, LBC showed a tendency toward an improved accuracy, although not to a significant degree (smear method: 43% vs. LBC: 65%; *P* = 0.139). The median diameter of the MPD was larger in HRS patients than in WF patients (10.0 mm vs. 5.0 mm; *P* < 0.001). Because MPD dilatation without obstruction in IPMN has been generally considered to be the result of mucus hypersecretion [31], HRS patients are expected to have more mucus than WF patients. Even using LBC with the BD SurePath, the improving effect of LBC was inadequate in cases rich in mucus, such as in HRS patients. However, in the clinical setting, it is important to detect malignant IPMNs in WF patients.

Enhancing MN > 5 mm negatively contributed to the accuracy of PJC (OR 0.23; *P* = 0.013). Of the 31 and 59 patients with and without enhancing MN > 5 mm, an accurate diagnosis was obtained in 17 (55%) and 41 (69%), respectively. The pathological diagnoses were as follows: L-IGD, 12; HGD, 5; and IC, 14 in patients with MN > 5 mm, and L-IGD, 35; HGD, 10; and IC, 14 in patients without MN > 5 mm. The proportion of malignant IPMN (HGD or IC) was larger in patients with enhancing MN > 5 mm than in those without enhancing MN > 5 mm (61% vs. 41%; *P* = 0.063). There was a bias between populations, and the sensitivity of PJC was low (28%) while the NPV was relatively high (60%). The accuracy was thus decreased in the patients with enhancing MN > 5 mm. However, enhancing MN > 5 mm itself is associated with malignant IPMN, so surgery should be recommended for patients with HRS, regardless of the results of PJC.

An elevated serum level of CA19–9 contributed to the accuracy of PJC (OR 0.24; *P* = 0.019). Of the 22 patients with an elevated level of CA19–9, an accurate diagnosis was obtained in 8 (36%). In contrast, of the 68 patients with normal levels of CA19–9, an accurate diagnosis was obtained in 50 (74%). Among the patients with elevated levels of CA19–9, 18 had malignant IPMN (HGD: 3 [14%], IC: 15 [68%]). In contrast, among the patients with normal levels of CA19–9, 25 had malignant IPMN (HGD: 12 [18%], IC: 13 [19%]). The proportion of IC was significantly larger in the patients with elevated levels of CA19–9 than in those with normal levels of CA19–9 (*P* < 0.0001). The sensitivity of PJC was low, but the NPV was high. Because of the bias between populations, the accuracy was decreased in the group with elevated levels of CA19–9. However, in the 22 patients with elevated levels of CA19–9, LBC improved the accuracy, although not to a significant degree (29 to 50%; *P* = 0.315). CA19–9 itself was also a useful marker for detecting IC derived from IPMN.

Yamakawa et al. reported that the sensitivity and accuracy of PJC for a malignant IPMN diagnosis were increased by a SPACE in IPMN patients [15]. Our study similarly showed that the sensitivity and accuracy of PJC were increased by a SPACE, although not to a significant degree (sensitivity: 26 to 50%; *P* = 0.301, accuracy: 62 to 83%; *P* = 0.142). Only 12 patients (13%) underwent a SPACE. Statistically significant differences might have been obtained if more patients had undergone a SPACE. Furthermore, of the 12 patients who underwent a SPACE, 11 were diagnosed by LBC, so it is necessary to consider the possibility of confounding by LBC.

Even using LBC methods, the sensitivity of PJC for malignant IPMN was not sufficient (40%), a PEP occurred in 12.5% of cases. Although the severity of PEP was only mild or moderate and there were no patients who needed to cancel or delay their surgery, we should consider the indication of PJC for surgical candidates. In this study, among 46 patients with HRS, 30 (65%) had malignant IPMN. Thus, the findings of HRS are an adequate indication for surgery, and PJC for patient with HRS may be unnecessary. Among patients with WF, all of patients with lymphadenopathy and a majority with increasing serum levels of CA19–9 were malignant IPMN (100, 82%). In contrast, 37% (15/41) of patients with a cyst size ≥3 cm had malignant lesions, and the cyst size was associated with the accuracy of PJC (OR: 4.41, *P* = 0.009). Given these results, patients with a cyst size of ≥3 cm without the above 2 WF factors are considered well-indicated for PJC with LBC.

This study has several limitations. First, this was a retrospective study. Not all procedures were performed by the same endoscopists, and not all cytology specimens were evaluated by the same pathologists. Second, this study had a selection bias, as the indications of PJC depended in part on the discretion of the attending physician. Third, the efficacy of SPACE was not evaluated sufficiently because of the lack of patients who underwent a SPACE. Fourth, there were relatively few patients with LBC among the WF patients.

## Conclusions

In conclusion, LBC improved the accuracy of PJC for malignant IPMN. Furthermore, in WF patients in particular, LBC proved useful for the accurate diagnosis.

## Data Availability

The datasets used and/or analyzed during the current study are available from the corresponding author on reasonable request.

## Abbreviations

PJC: pancreatic juice cytology
IPMN: intraductal papillary mucinous neoplasm
LBC: liquid-based cytology
HRS: high-risk stigmata
WF: worrisome features
MN: mural nodules
BD-IPMN: branch duct IPMN
ERCP: endoscopic retrograde cholangiopancreatography
PEP: post-ERCP pancreatitis
CT: computed tomography
EUS: endoscopic ultrasonography
MRCP: magnetic resonance cholangiopancreatography
L-IGD: low- to intermediate-grade dysplasia
HGD: high-grade dysplasia
IC: invasive carcinoma
SPACE: serial pancreatic juice aspiration cytological examination
ENPD: endoscopic naso-pancreatic drainage
IQR: interquartile range
MD-IPMN: main duct IPMN
MPD: main pancreatic duct
CA19–9: carbohydrate antigen 19–9
OR: odds ratio
NPV: negative predictive value
